# Influence of PhoPQ and PmrAB two component system alternations on colistin resistance from non-*mcr* colistin resistant clinical *E. Coli* strains

**DOI:** 10.1186/s12866-024-03259-8

**Published:** 2024-04-02

**Authors:** Ching-Hsun Wang, L. Kristopher Siu, Feng-Yee Chang, Yu-Kuo Tsai, Li-Yueh Huang, Jung-Chung Lin

**Affiliations:** 1 Division of Infectious Diseases and Tropical Medicine, Department of Internal Medicine, Tri-Service General Hospital, National Defense Medical Center, No. 325, Section 2, Cheng-Kung Road, Neihu 114, Taipei, Taiwan; 2 Institute of Infectious Diseases and Vaccinology, National Health Research Institutes, Miaoli, Taiwan

**Keywords:** Colistin, Resistance, *E. Coli*, Chromosome, PhoP, MgrB, PmrB

## Abstract

**Background:**

The current understanding of acquired chromosomal colistin resistance mechanisms in *Enterobacterales* primarily involves the disruption of the upstream PmrAB and PhoPQ two-component system (TCS) control caused by mutations in the regulatory genes. Interestingly, previous studies have yielded conflicting results regarding the interaction of regulatory genes related to colistin resistance in *Escherichia coli*, specifically those surrounding PhoPQ and PmrAB TCS.

**Results:**

In our study, we focused on two clinical non-*mcr* colistin-resistant strains of *E. coli*, TSAREC02 and TSAREC03, to gain a better understanding of their resistance mechanisms. Upon analysis, we discovered that TSAREC02 had a deletion (Δ27–45) in MgrB, as well as substitutions (G206R, Y222H) in PmrB. On the other hand, TSAREC03 exhibited a long deletion (Δ84–224) in PhoP, along with substitutions (M1I, L14P, P178S, T235N) in PmrB. We employed recombinant DNA techniques to explore the interaction between the PhoPQ and PmrAB two-component systems (TCSs) and examine the impact of the mutated *phoPQ* and *pmrB* genes on the minimum inhibitory concentrations (MICs) of colistin. We observed significant changes in the expression of the *pmrD* gene, which encodes a connector protein regulated by the PhoPQ TCS, in the TSAREC02 wild-type (WT)-*mgrB* replacement mutant and the TSAREC03 WT-*phoP* replacement mutant, compared to their respective parental strains. However, the expressions of *pmrB*/*pmrA*, which reflect PmrAB TCS activity, and the colistin MICs remained unchanged. In contrast, the colistin MICs and *pmrB*/*pmrA* expression levels were significantly reduced in the *pmrB* deletion mutants from both TSAREC02 and TSAREC03, compared to their parental strains. Moreover, we were able to restore colistin resistance and the expressions of *pmrB*/*pmrA* by transforming a plasmid containing the parental mutated *pmrB* back into the TSAREC02 and TSAREC03 mutants, respectively.

**Conclusion:**

While additional data from clinical *E. coli* isolates are necessary to validate whether our findings could be broadly applied to the *E. coli* population, our study illuminates distinct regulatory pathway interactions involving colistin resistance in *E. coli* compared to other species of *Enterobacterales.* The added information provided by our study contribute to a deeper understanding of the complex pathway interactions within *Enterobacterales*.

**Supplementary Information:**

The online version contains supplementary material available at 10.1186/s12866-024-03259-8.

## Background

The global emergence of colistin-resistant *Enterobacterales* in humans and animals has been observed in recent years owing to extensive colistin use in human and veterinary medicine [1]. Both chromosome- and plasmid-mediated colistin resistance mechanisms have been reported among colistin-resistant *Enterobacterales* [2]. While chromosome-encoded resistance mechanisms cannot be transferred between organisms, mutations in the core genome may lead to high genetic stability. This situation can pose a significant health concern when chromosomal mutations accumulate in major human pathogenic bacteria [3, 4]. The current understanding of acquired chromosomal colistin resistance mechanisms in *Enterobacterales* is primarily associated with the upregulation of the *pmrHFIJKLM* and *pmrCAB* operons leading to the enhanced modification of lipopolysaccharides (LPS) and development of colistin resistance [1]. This resistance mechanism is believed to be linked to disruptions in the upstream PmrAB and PhoPQ two-component systems (TCSs) controls. Critical genetic changes in the regulatory genes of these systems play an important role in the development of colistin resistance [5–11]. Notably, the interaction of regulatory genes surrounding PhoPQ and PmrAB TCSs related to colistin resistance varies across species of *Enterobacterales* [12, 13]. In the case of *Escherichia coli*, regulatory networks controlling the PhoPQ and PmrAB TCSs related to colistin resistance still need to be elucidated as previous studies have yielded conflicting results [13, 14].

In our previous study, we collected 11 *mcr*-negative colistin-resistant *E. coli* strains to investigate their chromosomal resistance mechanisms. We successfully confirmed that the R81H substitution in PmrA contributed to colistin resistance in three isolates. In contrast, the exact resistance mechanism to colistin is yet to be determined in the remaining eight isolates (TSAREC02, TSAEC03, TSAREC05, TSAREC06, TSAREC07, TSAREC08, TSAREC10, and TSAREC37), all of which harbored substitutions in PmrB [15]. As PhoPQ TCS activation due to mutated regulatory genes conferring colistin resistance has been reported in *Klebsiella pneumoniae*, we inferred this might also be true for *E. coli* [10, 11]. After comparing the regulatory genes *mgrB*, *phoP* and *phoQ* from the remaining eight *E. coli* isolates with those from colistin-susceptible isolates, we uncovered additional unique amino acid deletions in MgrB and PhoP from TSAREC02 and TSAREC03, respectively. To further investigate and validate our hypothesis, we generated different mutants of TSAREC02 and TSAREC03 to assess the impact of the mutations in *mgrB* and *phoP* on colistin resistance. This aspect has not been previously addressed yet. Additionally, we re-evaluated the influence of the individual mutated *pmrB* genes from TSAREC02 and TSAREC03 on colistin resistance using the respective mutants of TSAREC02 and TSAREC03. This approach differed from the previous study which utilized the MG16655 mutant with PmrB deletion as recipients [15].

## Methods

### Bacterial strains, plasmids, and antimicrobial susceptibility testing

The clinical colistin-resistant strains TSAREC02 and TSAREC03 were obtained from a previously described nationwide surveillance program and their relevant antimicrobial susceptibility data were collected as previously reported [15]. To determine the colistin minimum inhibitory concentrations (MICs) and breakpoints in the current study, we adhered to the broth microdilution method according to the guidelines provided by the international susceptibility testing committee, specifically the European Committee on Antimicrobial Susceptibility Testing 2021. For further information on the *E. coli* strains, plasmids, and primers used in this study, please refer to Supplementary Table S1, S2, and S3, respectively.

### PCR (polymerase chain reaction amplification) and sequencing of genes involved in colistin resistance

In addition to *pmrA* and *pmrB*, the genes *phoP*, *phoQ*, and *mgrB* from TSAREC02 and TSAREC03 were amplified and sequenced using the appropriate primers (Supplementary Table S3) [16]. As described previously, the sequences of *phoP*, *phoQ*, and *mgrB* from TSAREC02 and TSAREC03 were compared to those of the wild type (WT) *E. coli* reference strain MG1655 and eight colistin-sensitive clinical *E. coli* strains (ECS01, ECS02, ECS03, ECS04, ECS05, ECS06, ECS07, and ECS08) to exclude possible synonymous polymorphisms. This enables the identification of any unique amino acid substitutions that may contribute to colistin resistance in TSAREC02 and TSAREC03. Sequence comparisons was analyzed on the National Center for Biotechnology Information website (www.ncbi.nlm.nih.gov). The Basic Local Alignment Search Tool was used for this analysis [15]. Protein Variation Effect Analyzer (https://www.jcvi.org/research/provean) software was used to predict whether the unique amino acid substitutions found in PhoP, PhoQ, and MgrB would affect the function of these proteins.

### Construction of the *mgrB* or *pmrB* deletion mutant of TSAREC02, as well as the *phoP* or *pmrB* deletion mutants of TSAREC03

The generation of the *mgrB* deletion mutant of TSAREC02 and the *phoP* deletion mutant of TSAREC03 were carried out utilizing plasmid-based gene knockout methods with a suicide vector called pUT-KB plasmid [17]. The protocol for generating these mutants followed a similar approach as previously described but with minor modifications [15]. For the creation of the *mgrB* deletion mutant from TSAREC02, a two-step PCR approach was employed. Two PCR products were initially generated using primer sets KO*mgrB*_F-1/KO*mgrB*_R-1 and KO*mgrB*_F-2/KO*mgrB*_R-2. These PCR products contained complementary ends that would later be used for overlap PCR. To generate the final deletion fragment, the two PCR products with complementary ends were combined as templates and amplified using KO*mgrB*_F-1 and KO*mgrB*_R-2 in an overlap PCR reaction. This resulted in the creation of a 1,667-bp fragment lacking the *mgrB* gene with 15 nucleotides complementary to a PfoI-digested linear pUT-KB plasmid on both sides. The resulting gel-purified PCR then were cloned into a PfoI-digested linear pUT-KB plasmid with the In-Fusion HD cloning kit (TaKaRa Bio, Japan) according to the manufacturer’ s instructions. Thereafter, the final plasmid was constructed named pUT-KB-KO*mgrB*. The plasmid pUT-KB-KO*mgrB* then was transformed into *E. coli* S17-1 λpir through the process of electroporation. Subsequently, this plasmid was mobilized into TSAREC02 through conjugation. The transconjugants were screened on Luria-Bertani (LB) agar supplemented with kanamycin (50 µg/mL) and colistin (4 µg/mL). The selected transconjugants then were further incubated in 20 mL of brain-heart infusion broth for 6 h without kanamycin. Subsequently, they were spread onto LB agar containing 10% w/v sucrose. After the double-crossover events had occurred, sucrose-resistant and kanamycin sensitive colonies were selected (indicating loss of the plasmid). The *mgrB* deletion mutants of TSAREC02, hereafter, was referred to as TSAREC02_Δ*mgrB*^Δ43–47^. As for *phoP* deletion mutant construction from TSAREC03, the process is slimier to the previous steps described for the *mgrB* deletion mutant, with the main difference being the primer sets used. The *phoP* deletion mutant therefore was referred to as TSAREC03_Δ*phoP*^Δ84–224^. The *pmrB* deletion mutants from TSAREC02 and TSAREC03 were constructed as our reported study with some modification [4]. As for the *pmrB* deletion mutant of TSAREC02/TSAREC03, the strain TSAREC02/TSAREC03 was used as the template. Two PCR products were created using the primer sets KO*pmrB*_XbaI-F/ KO*pmrB*-R and KO*pmrB*-F/ KO*pmrB*_BcuI-R. The two PCR products contained complementary ends and were mixed as a template for the subsequent overlap PCR step. Using the primers KO*pmrB*_XbaI-F and KO*pmrB*_BcuI-R, the mixed PCR products were amplified again, resulting in a fragment with a 1088 bp deletion in the *pmrB* gene through overlap PCR. The resulting PCR fragment was subjected to digestion with XbaI and BcuI enzymes. Simultaneously, the pUT-KB plasmid was also digested with XbaI and BcuI. The digested PCR fragment was then inserted into the digested pUT-KB plasmid, resulting in the construction of the pUT-KB-KO*pmrB* plasmid. Further steps involving the homologous recombination method to construct the *pmrB* deletion mutant of TSAREC02/TSAREC03 were the same as TSAREC02_Δ*mgrB*^Δ43–47^ and TSAREC03_Δ*phoP*^Δ84–224^. The *pmrB* deletion mutants from TSAREC02 and TSAREC03 were referred to as TSAREC02 _Δ*pmrB*^g616a, t618g, t664c^ and TSAREC03_Δ*pmrB*^g3c, t41c, c532t, c704a^, respectively.

### Construction of the TSAREC02 mutant with a WT-*mgrB* replacement and the TSAREC03 mutant with a WT-*phoP* replacement

The mutated *mgrB* in TSAEC02 and the mutated *phoP* in TSAREC03 were replaced with WT-*pmrB* and *phoP* from *E.coli* MG1655, respectively. DNA fragments of the *mgrB* and *phoP* genes from *E. coli* MG1655 were amplified via PCR using the primer sets KO*mgrB*_F-1/KO*mgrB*_R-2 for *mgrB* and KO*phoP*_F-1/KO*phoP*_R-2 for *phoP*. The generated PCR fragments were cloned into pUT-KB, resulting in pUT-KB-WT-*mgrB* and pUT-KB-WT-*phoP*, respectively. Using a previously described allelic exchange method with a plasmid pUT-KB-WT-*mgrB* and a pUT-KB-WT-*phoP*, a TSAREC02 mutant with WT-*mgrB* replacement (TSAREC02 WT-*mgrB* revertant) and TSAREC03 mutant with WT-*phoP* replacement (TSAREC03 WT-*phoP* revertant) were created from TSAREC02_Δ*mgrB*^Δ43–47^ and TSAREC03_Δ*phoP*^Δ84–224^, respectively [4].

### Complementation of different TSAREC02 and TSAREC03 mutants

The *pmrB* genes from TSAREC02 and TSAREC03 were amplified using PCR.

The amplified *pmrB* genes were then directly inserted into the pCRII-TOPO TA vector (Invitrogen, U.S.A). The insertion was done following the protocol provided by the manufacturer. The ligation reaction between the amplified *pmrB* genes and the pCRII-TOPO vector resulted in the creation of different pCRII-TOPO-derived plasmids. Each generated plasmid containing a specific variant of the *pmrB* gene were transformed into different TSAREC02 and TSAREC03 mutants via electroporation method. In the current study, to confirm accuracy the target gene with its franking regions in deletion, revertant and complemented mutants, several experiments were performed. First, we employed PCR method using specific primers (see Table S3) to amplify the region of interest. Subsequently, agarose gel electrophoresis was conducted to visualize the amplicons and confirm their sizes as expected. The expected amplicon sizes using primer pairs *mgrB*-F and *mgrB*-R from TSAREC02, TSAREC02_Δ*mgrB*^Δ43–47^,and TSAREC02 WT-*mgrB* revertant were 297 bp, 115 and 297 bp, respectively. The expected amplicon size using primer pairs *phoP-*F and *phoP-*R from TSAREC03, TSAREC03_Δ*phoP*^Δ84–224^ and TSAREC03 WT-*phoP* revertant were 920 bp, 250 bp, 920 bp, respectively. The expected amplicon sizes using the primer pairs *pmrB*-F and *pmrB*-R from TSAREC02 and TSAREC02 _Δ*pmrB*^g616a, t618g, t664c^ as well as TSAREC03 and TSAREC03_Δ*pmrB*^g3c, t41c, c532t, c704a^ were 1397 bp and 302 bp, respectively. Additionally, two amplicons with sizes of 302 and 1397 bp from complemented strains were anticipated. These amplicons serve as indicators: the 302 bp fragment suggests the presence of the deleted *pmrB* gene in the chromosome, while the 1397 bp fragment indicates the intact cloned *pmrB* gene in the plasmid transformed. Finally, to ensure the accuracy of the results, the amplicons were subjected to sequencing using sequencing primer for validation (see Table S3). The primer pairs M13-F and M13-R designed based on the sequence of the pCRII-TOPO TA vector (Invitrogen, U.S.A) were used to sequence the cloned *pmrB* from complemented strains.

### Real-time quantitative PCR

An increased expression of the *pmrHFIJKLM* operon in *E. coli* is associated with colistin resistance [1]. The expression of the *pmrK* gene, representative of *pmrHFIJKLM* operon expression, was measured via real-time quantitative PCR method as described previously [15]. Other genes regulating PmrAB and PhoPQ systems which may be related to colistin resistance were also measured. Briefly, Bacterial RNA and cDNA were obtained using an RNeasy mini kit (Qiagen, U.S.A) and Prime Script RT Master Mix (TaKaRa Bio, Japan) according to the manufacturer’s instructions. Expression levels of *pmrK*, *phoP*, *pmrD*, *pmrA* and *pmrB* were estimated using SYBR Green PCR Master Mix (Thermo Fisher Scientific, U.S.A). The *gapA* gene served as an endogenous reference for normalizing expression levels. Primer pairs used were according to previous studies and were listed in Supplementary Table S3 [8, 14, 18, 19]. Expression levels were calibrated against the baseline expression level of *E. coli* MG1655, and fold change in expression was calculated using the comparative threshold cycle method [20]. Data are expressed as the mean ± standard deviation of four independent experiments (performed in triplicate). A Student’s t-test was used for statistical analysis. The *p* values < 0.05 were considered statistically significant.

## Results

### Detection of *mgrB and phoP* mutations in colistin-resistant *E. Coli* strains TSAREC02 and TSAREC03, respectively

In addition to *pmrA* and *pmrB*, the genes *phoP*, *phoQ*, and *mgrB* from TSAREC02 and TSAREC03, which are thought to be involved in chromosomally encoded colistin resistance in *Enterobacterales*, were amplified and sequenced [16]. We firstly determined the full nucleotide sequences of *mgrB*, *phoP*, and *phoQ* from the TSAREC02 and TSAREC03 strains and compared them to *E. coli* strain MG1655 and eight previously described colistin-susceptible strains (ECS01 to ECS08) [4, 15]. Nucleotide variations in *mgrB*, *phoP*, and *phoQ* that produce amino acid substitutions only in TSAREC02, TSAREC03, as well as *pmrB*, and *pmrA* reported previously, were shown in Table 1 [15].

**Table 1:** Amino acid changes in PmrA/PmrB, PhoP/PhoQ^a^, and MgrB in colistin-resistant *E. coli* strains^a^

Strain	Substitution(s)^b^ in				
	MgrB	PhoQ	PhoP	PmrA	PmrB
TSAREC02	Δ43-47^c^				G206R, Y222H
TSAREC03			Δ84-224^d^		M1I, L14P, P178S, T235N

^a^Unique amino acid substitutions found only in colistin-resistant *E. coli* strains TSAREC02 and TSAREC03 after alignment with clinical colistin-susceptible *E. coli* isolates (ECS01 to ECS08) and MG1655(8).

^b^The one-letter code for amino acid designation was used

^c^Deleted amino acid sequence: QFIPW

^d^Deleted amino acid sequence: WQDKVEVLSAGADDYVTKPFHIEEVMARMQALMRRNSGLASQVISLPPFQVDLSRRELSINDEVIKLTAFEYTIMETLIRNNGKVVSKDSLMLQLYPDAELRESHTIDVLMGRLRKKIQAQYPQEVITTVRGQGYLFELR

In addition to the amino acid substitutions in PmrB, a deletion of five amino acid residues (QFIPW) in MgrB and a long deletion of 140 amino acid residues in PhoP were observed in TSAREC02 and TSAREC03, respectively. The deletion mutations in MgrB and PhoP were predicted to affect protein function using the Protein Variation Effect Analyzer software (https://www.jcvi.org/research/provean).

### The impact of *mgrB* mutations in TSAREC02 and *phoP* mutations in TSAREC03 on colistin resistance

**Fig. 1:**
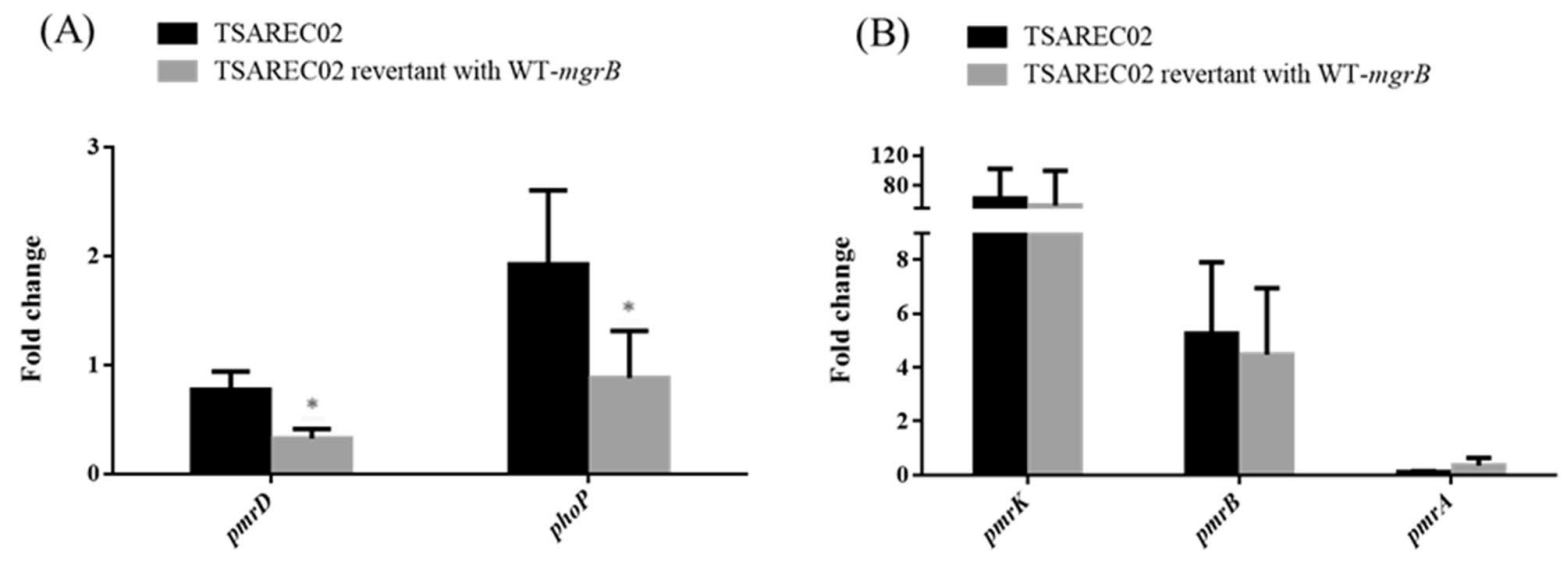
The mRNA expression levels of *pmrD*/*phoP* (**A**) and *pmrK*/*pmrB/pmrA* (**B**) in TSAREC02 and TSAREC02 WT-*mgrB* revertants. Values on the y axis are relative expression levels (fold change) normalized against levels in the colistin susceptible strain MG1655. * Significant differences compared to those in TSAREC02 (Student’s t-test, *p* < 0.05)

**Fig. 2:**
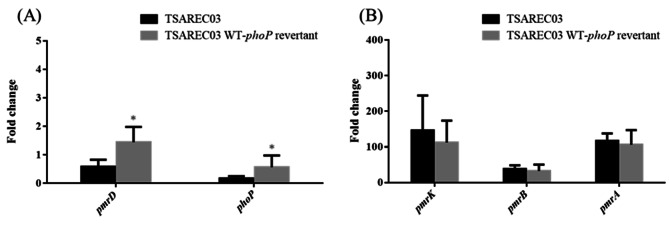
The mRNA expression levels of *pmrD*/*phoP* (**A**) and *pmrK*/*pmrB/pmrA* (**B**) in TSAREC03 and TSAREC03 WT-*phoP* revertants. Values on the y axis are relative expression levels (fold change) normalized against levels in the colistin susceptible strain MG1655. * Significant differences compared to those in TSAREC03 (Student’s t-test, *p* < 0.05)

Colistin resistance due to mutations in the regulatory genes controlling PhoPQ TCS has been reported in *K. pneumoniae* [10, 11]. To investigate the effect of *mgrB* and *phoP* mutations in TSAREC02 and TSAREC03 on colistin resistance, TSAREC02 mutants (including TSAREC02_Δ*mgrB*^Δ43–47^ and TSAREC02 WT-*mgrB* revertant) and TSAREC03 mutants (including TSAREC03_Δ*phoP*^Δ84–224^ and TSAREC03 WT-*phoP* revertant) were constructed. The amplicons from each constructs formed a distinct band with the expected size after agarose gel electrophoresis in Fig. S1 (A and B). Compared to TSAREC02, no change in colistin MIC values were observed in TSAREC02_Δ*mgrB*^Δ43–47^ and TSAREC02 WT-*mgrB* revertant (Table 2). Similarly, compared to TSAREC03, no change in colistin MIC values was noted in TSAREC03_Δ*phoP*^Δ84–224^ and TSAREC03 WT-*phoP* revertant (Table 3). The expression levels of *pmrD*, *phoP*, *pmrK*, *pmrA*, and *pmrB* in different mutants of TSAREC02 and TSAREC03 were shown in Tables 2 and 3; Figs. 1 and 2. As shown in Fig. 1; Table 2, *pmrD* and *phoP* expression levels decreased in the TSAREC02 WT-*mgrB* revertant compared to the TSAREC02 strain. However, the expression levels of *pmrK*, *pmrB*, and *pmrA* in the TSAREC02 WT-*mgrB* revertant were comparable to those in TSAREC02. As shown in Fig. 2; Table 3, the TSAREC03 WT-*phoP* revertant showed higher *pmrD* and *phoP* expressions compared to TSAREC03. Comparable expression levels of *pmrK, pmrA*, and *pmrB* were observed between TSAREC03 and the TSAREC03 WT-*phoP* revertant. Based on these results, it appears that *mgrB*/*phoP* mutations in TSAREC02/TSAREC03 contributing to PhoPQ alteration did not result in changes in PmrAB TCS activity that further influenced downstream *pmrHFIJKLM* expression levels and colistin MIC values.

**Table 2:** Colistin MICs and gene expression levels in different *E. coli* TSAREC02 mutants

Strain	Chromosomal mgrB status^a,b^	Chromosomal pmrB status	Episomal pmrB status	Relative expression level (mean ± SD)					Colistin MIC(µg/mL)
				phoP	pmrD	pmrA	pmrB	pmrK	
MG1655	WT	WT	None	1	1	1	1	1	0.5
TSAREC02	c127t (premature termination)	g616a, t618g, t664c (G206R, Y222H.)	None	1.92 ± 0.68	0.77 ± 0.17	0.11 ± 0.03	5.26 ± 2.65	63.04 ± 39.34	16
TSAREC02_Δ*mgrB*^Δ43–47^	Deleted	g616a, t618g, t664c (G206R, Y222H.)	None	ND	ND	ND	ND	ND	16
TSAREC02 WT-*mgrB* revertant	WT^b^	g616a, t618g, t664c (G206R, Y222H.)	None	0.88 ± 0.43	0.32 ± 0.09	0.35 ± 0.28	4.48 ± 2.47	53,65 ± 46.35	16
TSAREC02 _Δ*pmrB*^g616a, t618g, t664c^	c127t (premature termination)	Deleted	None	ND	ND	0.01 ± 0.001	< 0.01	3.08 ± 0.88	0.5
TSAREC02 _Δ*pmrB*^g616a, t618g, t664c^ (pCRII-TOPO*pmrB*^g616a, t618g, t664c^)	c127t (premature termination)	Deleted	g616a, t618g, t664c (G206R, Y222H.)	ND	ND	0.12 ± 0.03	18.54 ± 4.22	83.41 ± 22.63	16

MIC, minimum inhibitory concentration; ND, not determined

^a^The one-letter code for the amino acid designation is used

^b^WT, wild type indicating that the sequence was identical to that of the colistin-susceptible *E.coli* strain MG1655 or ESC01 to ECS08.^8^

**Table 3:** Colistin MICs and gene expression levels in different *E. coli* TSAREC03 mutants

Strain	Chromosomal phoP status^a, b^	Chromosomal pmrB status	Episomal pmrB status	Relative expression level (mean ± SD)					Colistin MIC (µg/mL)
				phoP	pmrD	pmrA	pmrB	pmrK	
MG1655	WT	WT	None	1	1	1	1	1	0.5
TSAREC03	g252a (premature termination)	g3c, t41c, c532t, c704a (M1I, L14P,P178S,T235N)	None	0.17 ± 0.08	0.59 ± 0.24	116.79 ± 21.12	38.18 ± 10.46	146.32 ± 98.16	8
TSAREC03_Δ*phoP*^Δ84–224^	Deleted	g3c, t41c, c532t, c704a (M1I, L14P,P178S,T235N)	None	ND	ND	ND	ND	ND	8
TSAREC03 WT-*phoP* revertant	WT	g3c, t41c, c532t, c704a (M1I, L14P,P178S,T235N)	None	0.56 ± 0.41	1.44 ± 0.54	106.04 ± 41.17	32.53 ± 17.84	112.03 ± 61.87	8
TSAREC03_Δ*pmrB*^g3c, t41c, c532t, c704a^	g252a (premature termination)	Deleted	None	ND	ND	4.66 ± 0.56	< 0.01	2.74 ± 0.71	0.25
TSAREC03 _Δ*pmrB*^g3c, t41c, c532t, c704a^ (pCRII-TOPO*pmrB*^g3c, t41c, c532t, c704a^)	g252a (premature termination)	Deleted	g3c, t41c, c532t, c704a (M1I, L14P,P178S,T235N)	ND	ND	157.00 ± 43.61	66.52 ± 44.80	251.70 ± 133.80	8

MIC, minimum inhibitory concentration

^a^The one-letter code was used for amino acid designation

^b^WT, wild type, indicating that the sequence was identical to that of the colistin-susceptible *E.coli* strains MG1655 or ESC01 to ECS08

### The impact of *pmrB* mutations in TSAREC02 and TSAREC03 on colistin resistance

**Fig. 3:**
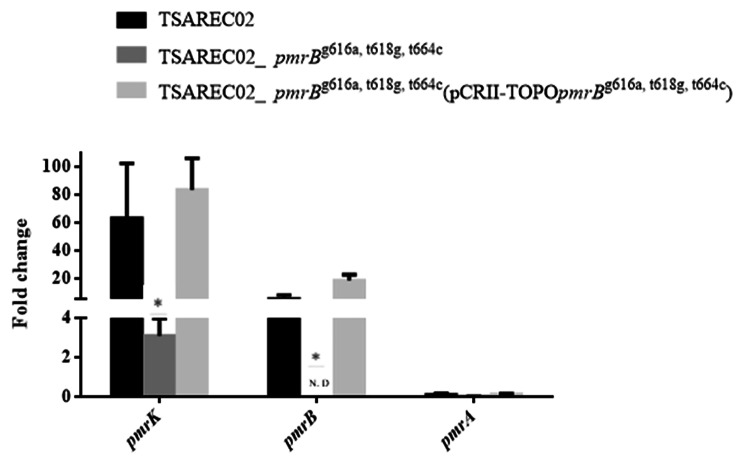
The mRNA expression levels of *pmrB*/*pmrK* in different TSAREC02 mutants. Values on the y axis are relative expression levels (fold change) normalized against levels in the colistin susceptible strain MG1655. * Significant differences compared to those in TSAREC02 (Student’s t-test, *P* < 0.05). N.D., non-detectable or expression level < 0.01

We re-evaluated the effect of amino acid substitutions in PmrB from TSAREC02 and TSAREC03 on colistin resistance using different TSAREC02 and TSAREC03 mutants. The amplicons from different TSAREC02 and TSAREC03 mutants exhibited a distinct band with the expected sizes following agarose gel electrophoresis as shown in Fig. S1 (C and D). As shown in Table 2, the TSAREC02 mutant with a *pmrB* deletion showed colistin susceptibility, with an MIC of 0.5 µg/mL. Following transformation with a recombinant plasmid containing a parental mutated *pmrB*, the complemented strain TSAREC02 _Δ*pmrB*^g616a, t618g, t664c^ (pCRII-TOPO*pmrB*^g616a, t618g, t664c^), exhibited restored colistin resistance (colistin MIC 16 µg/mL). Consistent with the changes in colistin MIC levels, the expression levels of *pmrA*, *pmrB*, and *pmrK* increased in the complemented strains TSAREC02 _Δ*pmrB*^g616a, t618g, t664c^ (pCRII-TOPO*pmrB*^g616a, t618g, t664c^) compared to TSAREC02 _Δ*pmrB*^g616a, t618g, t664c^ (Table 2; Fig. 3).

**Fig. 4:**
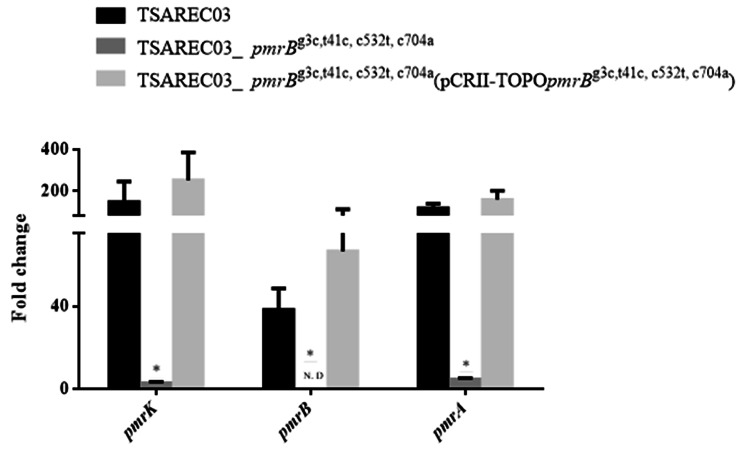
The mRNA expression levels of *pmrB*/*pmrK* in TSAREC03 mutants. Values on the y axis are relative expression levels (fold change) normalized against levels in the colistin susceptible strain MG1655. * Significant differences compared to those in TSAREC03 (Student’s t-test, *P* < 0.05). N.D., non-detectable or expression

In Table 3, the TSAREC03 mutant with a *pmrB* deletion showed colistin susceptibility (MIC = 0.25 µg/mL) and restored colistin resistance (MIC = 8 µg/mL) after transformation with a recombinant plasmid containing a parental mutated *pmrB*. Moreover, the TSAREC03 mutant with a *pmrB* deletion revealed increased expression levels of *pmrA, pmrB*, and *pmrK* after the introduction of a plasmid carrying the parental mutated *pmrB*, consistent with the increased colistin MIC values (Fig. 4). Taken together, these results confirm that amino acid substitutions in PmrB confer colistin resistance to TSAREC02 and TSAREC03.

### Nucleotide sequence accession numbers

The complete nucleotide sequences of mutated *mgrB* and *pmrB* from TSAREC02 and mutated *phoP* and *pmrB* from TSAREC03 were deposited in the GenBank nucleotide database under accession numbers MT597405, MT597406, MT597417, and MT597407, respectively.

## Discussion

Studies have reported differences in regulatory gene interactions involving the PmrA/PmrB and PhoP/PhoQ TCSs which contribute to colistin resistance in different species of *Enterobacterales*. This suggests a more complex network of interactions than what was previously understood [9, 12, 13, 21]. The PmrA/PmrB TCS in *Salmonella enterica* and *K. pneumoniae* is known to responds to a signal that controls the PhoP/PhoQ TCS through the involvement of PmrD [22, 23]. However, it is important to note that while *E. coli* also encodes a homolog of PmrD, the study of experimentally confirmed mutated genes associated with colistin resistance in *E. coli* has been primarily limited to *pmrAB* [4, 6, 8, 15, 24]. Previous studies have reported amino acid substitutions in the PhoPQ and MgrB proteins of *E. coli* in association with colistin resistance. However, none of these reported substitutions have been experimentally validated for their specific contribution to colistin resistance [25–27]. Conflicting results regarding the influence of PmrD on PmrAB activity and colistin resistance have been reported in two studies. A study by Winfield et al. revealed that the PmrAB TCS was not affected by PhoPQ TCS; in contrast, a study by Rubin et al. showed the opposite result that the PmrAB TCS could be indirectly activated by the upregulated PhoPQ TCS via PmrD [13, 14]. The interaction between PmrAB and the PhoPQ TCS was investigated in these two studies using different strains of *E. coli.* Winfield et al. used *E. coli* MG1655 whereas Rubin et al. used the closely related K-12 *E. coli* strain W3110. Both studies examined this interaction under varying Mg^2+^ concentrations, comparing low versus high Mg^2+^ environments. Since the phosphatase activity of the PmrB protein plays a major role in PmrAB TCS activity and colistin resistance, differences in the strains or unidentified environmental stimuli that affect PmrB phosphatase activity in the *E.coli* isolates in the two studies may account for the discrepancy in their results [28]. In the current study, we used clinical strains TSAREC02 and TSAREC03 with *mgrB* and *phoP* mutations, respectively, to evaluate the influence of PhoPQ alterations on PmrAB TCS and colistin resistance. Both studied isolates, TSAREC02 and TSAREC03, with colistin resistance were of clinical origin from humans. Moreover, we used colistin MIC levels to indicate colistin resistance phenotypes of studied isolates. Therefore, our study provides insights into the real-world scenarios of colistin resistance in human clinical settings. This enhances the clinical relevance and applicability of our results when compared to previous reports [13, 14]. Considering MgrB as a negative regulator and PhoP as a positive regulator in controlling PhoPQ TCS activity, we can observe increased *phoP* expressions in TSAREC02 with mutated *mgrB* and decreased *phoP* expressions in TSAER03 with mutated *phoP* [10, 11]. As expected, *phoP* expressions changed significantly in TSAREC02 revertant with WT-*mgrB*, and in TSAER03 revertant with WT-*phoP.* Moreover, *pmrD* expressions regulated by PhoPQ TCS changing significantly were also observed (Figs. 1 and 2). Nevertheless, expressions of *pmrAB* and downstream regulatory gene *pmrK* did not change significantly between strains TSAREC02 and TSAREC02 WT-*mgrB* revertant, as well as TSAREC03 and TSAREC03 WT-*phoP* revertant. Consequently, colistin MICs remained unchanged among mutants of TSAREC02 and TSAREC03. The results suggested that PhoPQ TCS alterations due to mutated *phoP* or *mgrB* that led to *pmrD* expressions change do not contribute to PmrAB TCS activity alternation and subsequently colistin MICs changes. No change in colistin MIC values observed in the TSAREC02 mutant, TSAREC02_Δ*mgrB*^Δ43–47^, and TSAREC03 mutant, TSAREC03_Δ*phoP*^Δ84–224^, further support such speculation. These results may explain why clinical colistin-resistant isolates resulting from PhoPQ activation due to a mutated regulatory gene have not been reported yet.

Current experimentally confirmed results regarding chromosomal colistin resistance from a few clinical or laboratory strains showing that PmrB modification is the primary mechanism for colistin resistance in *E. col*i [6, 18, 24]. In our previous study, eight colistin-resistant *E.coli* strains harbored unique amino acid substitutions in PmrB. However, no change in the colistin MICs was observed after a plasmid containing each mutated *pmrB* from the respective colistin-resistant strain was cloned and expressed in the MG1655 mutant with *pmrB* deletion. These results showed that mutated *pmrB* genes from *E. coli* isolates were not associated with colistin resistance [15]. The surprising result could stem from either the activation of operon*s pmrHFIJKLM* and *pmrCAB* in a PmrAB-independent manner or an unknown factor in strain MG1655 that interfered with the effect of the cloned *pmrB*-carrying plasmid. In the current study, we re-evaluated the effect of *pmrB* mutations in TSAREC02 and TSAREC03 on colistin resistance using different validation methods. The mutated *pmrB* from TSAREC02 and TSAREC03 was cloned into plasmids and expressed in TSAREC02 and TSAREC03 mutants with *pmrB* deletion, respectively. Therefore, confounding factors from the genomic backgrounds of different *E. coli* strains could be eliminated. The results of the present study indicate that *pmrB* mutation is an independent factor for colistin resistance in TSAREC02 and TSAREC03 via increased PmrAB TCS activity. Moreover, the result support our speculation that some unknown interfering factors in the genomic background of *E.coli* MG1655 may influence the effect of the cloned mutated *pmrB* on colistin resistance [15]. Based on the results demonstrated here, we inferred that the colistin resistance mechanism among the remaining six *E. coli* isolates collected in a previous study may still result from the unique *pmrB* mutation they harbored [15]. Further investigations of these strains using a method similar to that used in the current study are underway. Moreover, significantly different expression levels of *pmrA* between TSAREC02 and TSAREC03 strains were observed. Since PmrA (also known as BasR) has interaction with many pathways reported before based up on STRING database (https://version-11-5.string-db.org/network/511145.b4113), different *pmrA* expression levels between 2 strains may be related to different regulative activity of interactive pathways. Since no mutations in *pmrA* from both strains and both complemented strains with mutated *prmB* reached comparable colistin MIC levels to the original clinical strains, we speculated that different *pmrA* expressions on both strains had no influence on colistin resistance.

## Conclusion

Although *E. coli* is a significant human pathogen within *Enterobacterales*, studies about chromosome-encoded pathway interactions involving colistin resistance is less described due to its rarity in clinical origin. Our study, based on two clinical colistin resistant *E. coli* isolates, elucidated the interaction between the PhoPQ and PmrAB TCs and their influence on colistin resistance. Our results demonstrated that alternations in the PhoPQ TC, caused by mutated regulatory genes, did not influence colistin resistance. Instead, mutations in the *pmrB* gene were identified as the key factor contributing to colistin resistance in both studied clinical *E. coli* isolates. For new drug development, proper target selection is crucial. Complex regulatory pathway interactions involving colistin resistance control were observed among different species of *Enterobacterales*. This complexity makes it challenging to select universal targets that cover all olistin resistant *Enterobacterales* during drug development. Although our study was limited to only two clinical isolates and the results may not be generalizable to the entire *E. coli* population, it provides valuable insights into the mechanisms underlying colistin resistance. These findings have the potential to contribute significantly to the development of new drugs targeting emerging colistin-resistant *Enterobacterales.*

### Electronic supplementary material

Below is the link to the electronic supplementary material.

Supplementary Material 1

Supplementary Material 2

Supplementary Material 3

Supplementary Material 4

Supplementary Material 5

## Data Availability

No datasets were generated or analysed during the current study.

## Abbreviations

LPS: Lipopolysaccharides
TCS: Two-component system
PCR: Polymerase chain reaction
MIC: Minimum inhibitory concentration
WT: Wild type
LB: Luria-Bertani
