# Supplementary material for: Influence of PhoPQ and PmrAB two component system alternations on colistin resistance from non-mcr colistin resistant clinical E. Coli strains
Source: BMC Microbiol. 2024 Apr 2;24:109. doi: 10.1186/s12866-024-03259-8 (PMC10986093; doi:10.1186/s12866-024-03259-8)
Supplement: Supplementary file 1 — Supplementary Material 1 [file 12866_2024_3259_MOESM1_ESM.doc]

Table S1. *Escherichia coli* strains used in this study.

| Strain | Relevant characteristic(s) | Source / Reference |
| --- | --- | --- |
| MG1655 | Wild type | ATCC |
| ATCC 25922 | Wild type | ATCC |
| S17-1λpir | hsdR recA pro RP4-2 (Tc::Mu; Km::Tn7) (λpir) | [4] |
| TSAREC02 | Clinical isolate with colistin resistance | [15] |
| TSAREC03 | Clinical isolate with colistin resistance | [15] |
| ECS01 | Clinical isolate susceptible to colistin, novel ST | [4] |
| ECS02 | Clinical isolate susceptible to colistin, ST73 | [4] |
| ECS03 | Clinical isolate susceptible to colistin, ST131 | [4] |
| ECS04 | Clinical isolate susceptible to colistin, ST69 | [4] |
| ECS05 | Clinical isolate susceptible to colistin, ST38 | [4] |
| ECS06 | Clinical isolate susceptible to colistin, ST73 | [4] |
| ECS07 | Clinical isolate susceptible to colistin, ST1193 | [4] |
| ECS08 | Clinical isolate susceptible to colistin, ST131 | [4] |
| TSAREC02_Δ*mgrB*^Δ43-47^ | Complete deletion of the *mgrB*^Δ43-47^ locus in TSAREC02 | This study |
| TSAREC02 WT-*mgrB* revertant | TSAREC02 background; strain MG1655 WT-*mgrB* replacement | This study |
| TSAREC02_Δ*pmrB*^g616a, t618g, t664c^ | 1088-bp deletion in the *pmrB*^g616a, t618g, t664c^ locus of TSAREC02 | This study |
| TSAREC02 _Δ*pmrB*^g616a, t618g, t664c^  (pCRII-TOPO*pmrB*^g616a, t618g, t664c^) | TSAREC02_Δ*pmrB*^g616a, t618g, t664c^ complemented with  pCRII-TOPO*pmrB*^g616a, t618g, t664c^ | This study |
| TSAREC03_Δ*phoP*^Δ84-224^ | 567-bp deletion in the *phoP* locus of TSAREC03 | This study |
| TSAREC03 WT-*phoP* revertant | TSAREC03 background; strain MG1655 WT-*phoP* replacement | This study |
| TSAREC03_Δ *pmrB*^g3c, t41c, c532t, c704a^ | 1088-bp deletion in the *pmrB*^g3c, t41c, c532t, c704a^ locus of TSAREC03 | This study |
| TSAREC03 _Δ *pmrB*^g3c, t41c, c532t, c704a^ (pCRII-TOPO*pmrB*^g3c, t41c, c532t, c704a^) | TSAREC02_Δ*pmrB*^g3c, t41c, c532t, c704a^ complemented with  pCRII-TOPO*pmrB*^g3c, t41c, c532t, c704a^ | This study |
|  | | |
|  | | |

| Table S2. Plasmids used in this study. | | |
| --- | --- | --- |
| Plasmid | Relevant characteristic(s) | Source / Reference |
| pCRII-TOPO | TOPO cloning vector; Amp^r^ Kan^r^ | Invitrogen, U.S.A |
| pUT-KB | Suicide vector; Kan^r^ | [17] |
| pCRII-TOPO*pmrB*^g616a, t618g, t664c^ | The *pmrB* ^g616a, t618g, t664c^ gene from *E. coli* TSAREC02 cloned into pCRII-TOPO | This study |
| pCRII-TOPO*pmrB*^g3c, t41c, c532t, c704a^ | The *pmrB*^g3c, t41c, c532t, c704a^ gene from *E. coli* TSAREC03 cloned into pCRII-TOPO | This study |
| pUT-KB-KO*pmrB*^g616a, t618g, t664c^ | 1,875-bp fragment containing an 1088-bp deletion in *pmrB*^g616a, t618g, t664c^ from  TSAREC02 cloned into pUT-KB | This study |
| pUT-KB-KO*pmrB*^g3c, t41c, c532t, c704a^ | 1,875-bp fragment containing an 1088-bp deletion in *pmrB*^g3c, t41c, c532t, c704a^ from  TSAREC03 cloned into pUT-KB | This study |
| pUT-KB-KO*mgrB* | 1,667-bp fragment with no *mgrB* from TSAREC02 cloned into pUT-KB | This study |
| pUT-KB-WT-*mgrB* | 1,849-bp fragment containing an entire WT-*mgrB* from MG1655 cloned into pUT-KB | This study |
| pUT-KB-KO*phoP* | 1,927-bp fragment containing an 567-bp deletion in *phoP* from TSAREC02  cloned into pUT-KB | This study |
| pUT-KB- WT-*phoP* | 2,604-bp fragment containing an entire WT-*phoP* from MG1655 cloned into pUT-KB | This study |

Amp^r^, resistance to ampicillin; Kan^r^, resistance to kanamycin.

Table S3. Primers used in this study.

| Primer name | Sequence (5′-3′) | Reference |
| --- | --- | --- |
| **PCR scanning and DNA sequencing** | | |
| *mgrB*-F | AAGGTAGGTGAAACGGAGATT | [16] |
| *mgrB*-R | CCGATACAACCAAAGACGC |
| *phoP*-F | ATGGCGATGCTGTCCG | [16] |
| *phoP*-R | TCCGTAGGCAAGCGAAA |
| *phoQ-*F | GCAAAGTGGTCAGCAAAGA | [16] |
| *phoQ-R* | AATCGGGCCAGTTAAGAGT |
| *pmrB-F* | CCAACACCCTGGAAGTGC | [16] |
| *pmrB-R* | TGATGAATAAGCTGAAACGG |
|  | **DNA sequencing the gene *pmrB* in pCRII-TOPO*pmrB*^g616a, t618g, t664c^ and pCRII-TOPO*pmrB*^g3c, t41c, c532t, c704a^** |  |
| M13*-*F | GTAAAACGACGGCCAG | Invitrogen, U.S.A |
| M13*-*R | CAGGAAACAGCTATGAC |
| **Mutagenesis** | | |
| KO*mgrB*_F-1 | ACATGCAGCTCCCGGAGTGGAGTATAAACGCAGT^a^ | This study |
| KO*mgrB*_R-1 | TCCAATCTCCGTTTCACCTACCTTATGTCA^b^ |
| KO*mgrB*_F-2 | TGACATAAGGTAGGTGAAACGGAGATTGGAAGTGGTAGAATAGCGCCTC^b^ | This study |
| KO*mgrB*_R-2 | GTGACCGTCTCCGGGATGCTTAACCTTGCCAGTTC^a^ |
| KO*phoP*_F-1 | ACATGCAGCTCCCGGACACGCGGTTTCTGAATTC^a^ | This study |
| KO*phoP*_R-1 | AGCGGATAATCTTAATAAGCAGGCCGGACA^b^ |
| KO*phoP*_F-2 | TGTCCGGCCTGCTTATTAAGATTATCCGCTATTGATGTACTGATGGGACG^b^ | This study |
| KO*phoP*_R-2 | GTGACCGTCTCCGGGACTGGATTGAGCAATTCGC^a^ |
| KO*pmrB*_XbaI-F | AC***tctaga***CCCAACCTGCGACACCAATG ^c^ | [4] |
| KO*pmrB*-R | GCATCAGATTCAATTAGTTTTCCTCATTCG^b^ |
| KO*pmrB*-F | CGAATGAGGAAAACTAATTGAATCTGATGCTGCTGACCACCAGCACGCTG^b^ | [4] |
| KO*pmrB*_BcuI-R | AC***actagt***CGGTATGCTGTTTGTCCAGCC ^c^ |
| **Real-time RT-qPCR** |  |  |
| *pmrK*-F | TGCGGAAATCAGTCGAGAAATGC | [8] |
| *pmrK*-R | CGAAATAACGTAGCCCTAACAGATGG |
| *pmrB-*F | TCCCCTCGTATGACGAACTC | [18] |
| *pmrB-*R | TCATAATGTTGCTGCCTTGC |
| *pmrD-*F | ATGGAATGGCTGGTCAAAAA | [14] |
| *pmrD-*R | CATTCTGCAAAGGCGAGAGT |
| *pmrA-*F | CCTTTTGCGCTGGAAGAG | [18] |
| *pmrA-*R | TGGGCGTCAGAATCAACTC |
| *phoP-*F | CCGGTGCTGATGATTATGTG | [19] |
| *PhoP-*R | ATGACCTGTGAAGCCAGAC |
| *mgrB-*F | AGTTTCGATGGGTCGTTCTG | [19] |
| *mgrB-*R | TGATCGCACATCATGTTGAA |
| *gapA-*F | CGACAAATATGCTGGCCAGG | [8] |
| *gapA -*R | GTAGTAGCGTGAACGGTGGT |

^a^ The underlined sequences correspond to the overlapping region of the *Pfo*I-digested linear plasmid pUT-KB for in-fusion cloning.

^b^ The underlined sequences are complementary to the non-contiguous sequences and were used to introduce internal deletions via overlap PCR.

^c^ Restriction sites designed in the oligonucleotide are in boldface italics.
