# Supplementary figures and images for: Influence of PhoPQ and PmrAB two component system alternations on colistin resistance from non-mcr colistin resistant clinical E. Coli strains
Source: BMC Microbiol. 2024 Apr 2;24:109. doi: 10.1186/s12866-024-03259-8 (PMC10986093; doi:10.1186/s12866-024-03259-8)

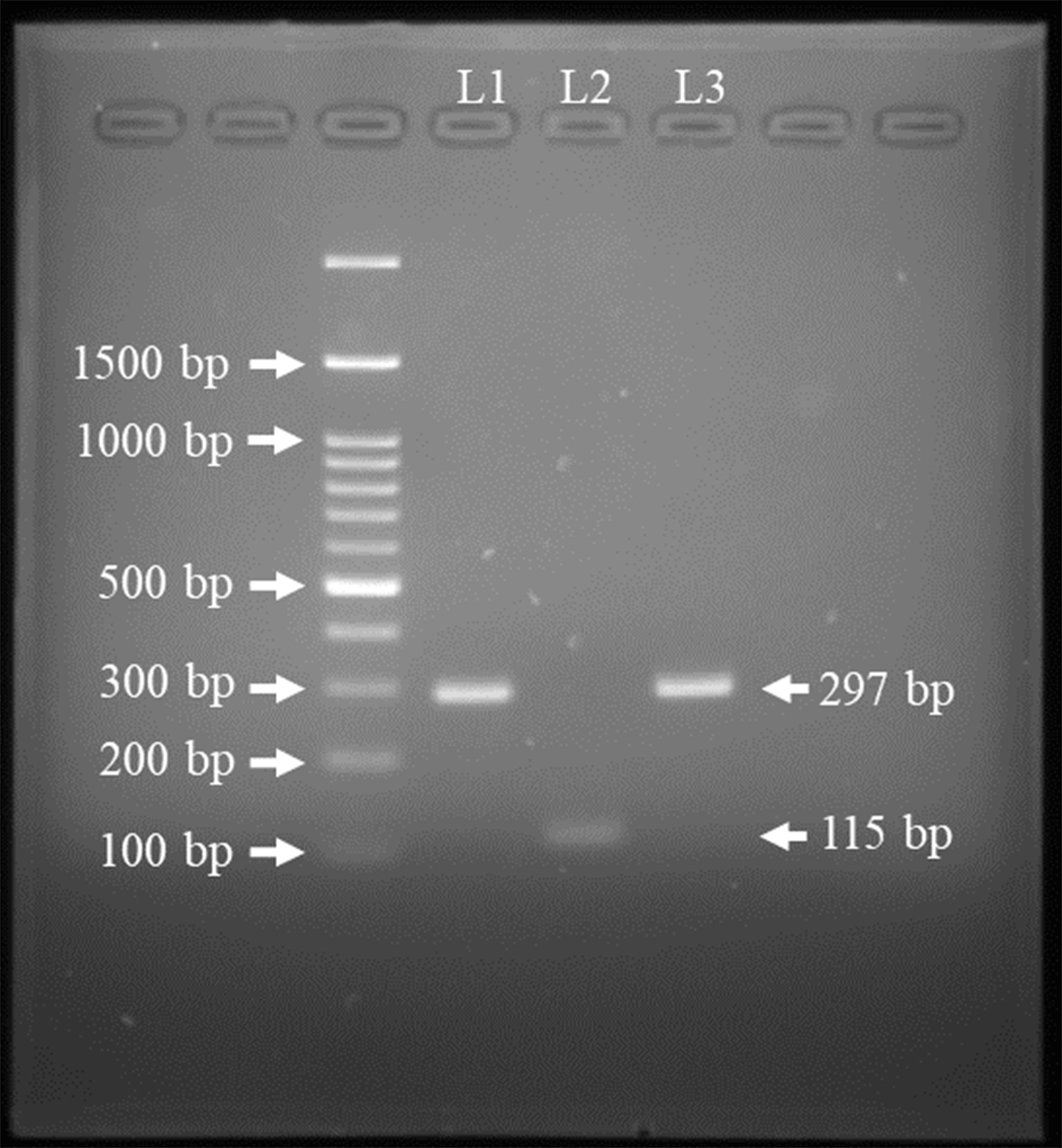

Supplement: Supplementary file 2 — Supplementary Material 2 [file 12866_2024_3259_MOESM2_ESM.tif]

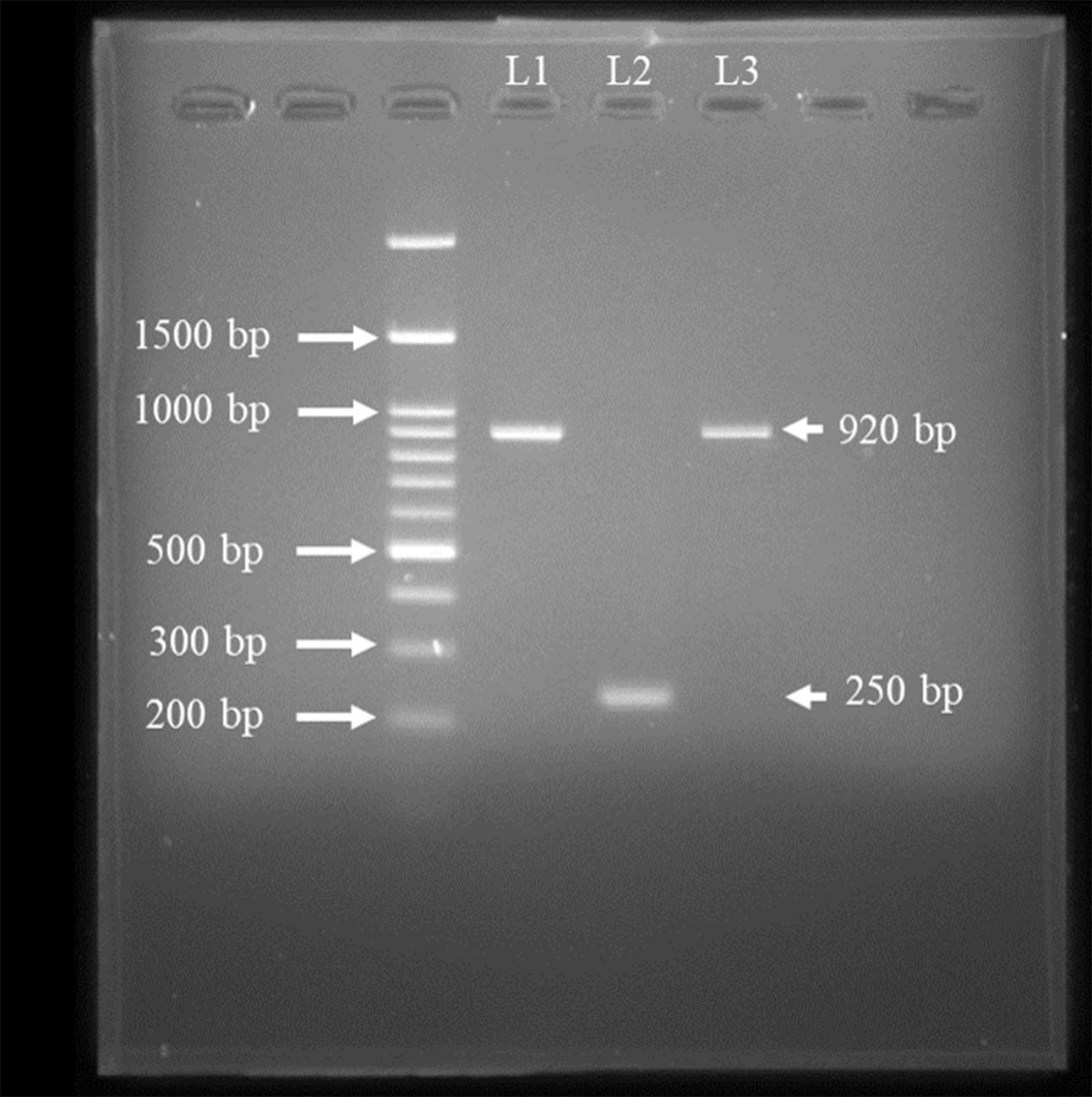

Supplement: Supplementary file 3 — Supplementary Material 3 [file 12866_2024_3259_MOESM3_ESM.tif]

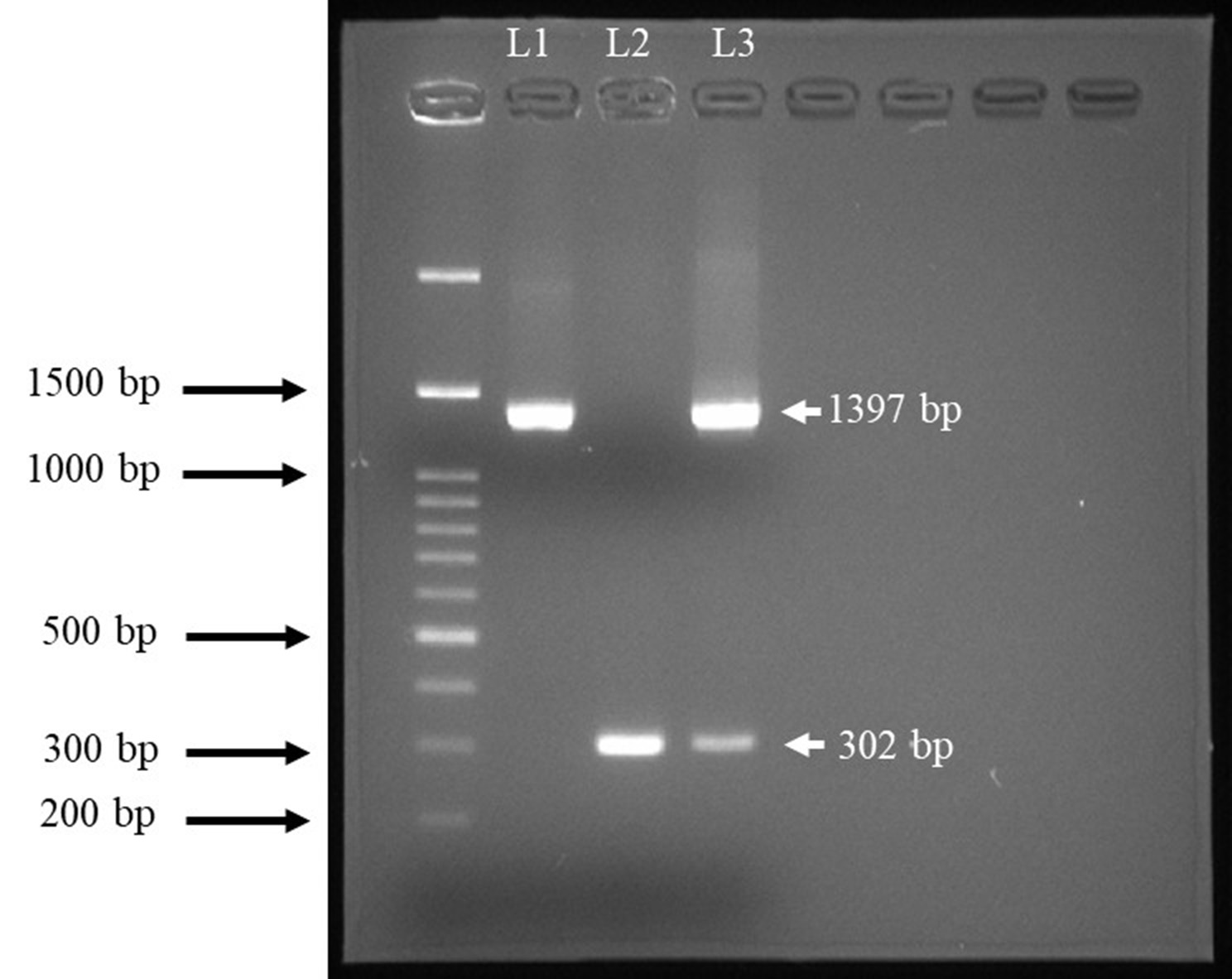

Supplement: Supplementary file 4 — Supplementary Material 4 [file 12866_2024_3259_MOESM4_ESM.jpg]

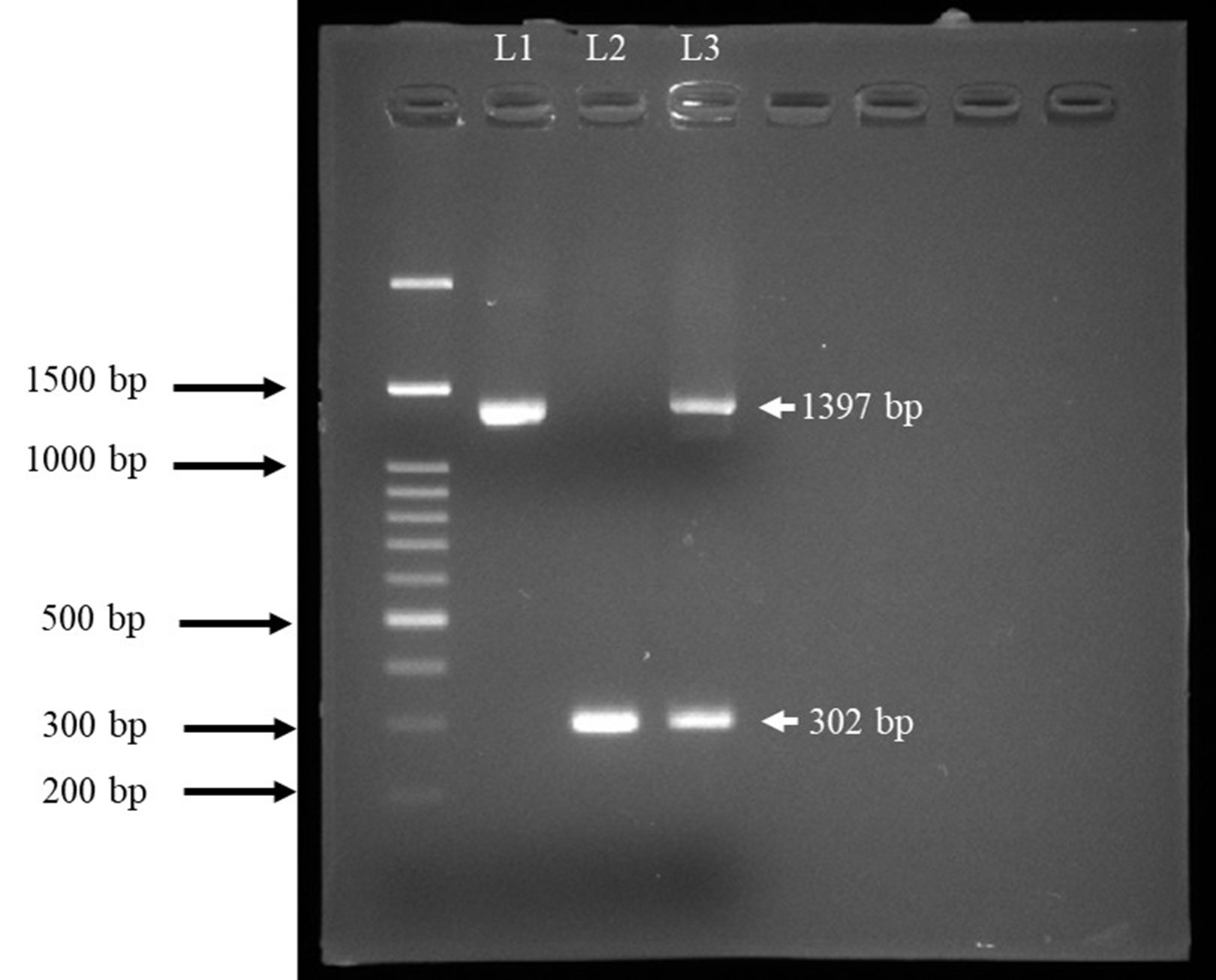

Supplement: Supplementary file 5 — Supplementary Material 5 [file 12866_2024_3259_MOESM5_ESM.jpg]
